# Decreased myometrial p160 ROCK-1 expression in obese women at term pregnancy 

**DOI:** 10.1186/1477-7827-11-79

**Published:** 2013-08-15

**Authors:** Margaret O’Brien, Shawna Carbin, John J Morrison, Terry J Smith

**Affiliations:** 1 Department of Obstetrics and Gynaecology, Clinical Sciences Institute, University College Hospital Galway, Galway, Ireland; 2 National Centre for Biomedical and Engineering Science, Orbsen Building, National University of Ireland, Galway University Road, Galway, Ireland

**Keywords:** p160 ROCK 1 protein, Myometrium, Obesity, Term pregnancy

## Abstract

**Background:**

Obesity is becoming an increasing problem in obstetric practice; it has led to an increase in the risk of caesarean delivery, prolonged pregnancy and dysfunctional labour. It has been postulated that many of these problems are as a result of abnormal myometrial contractility. The RhoA/Rho kinase pathway is involved in calcium sensitisation in the myometrium during labour and contributes to the phosphorylation of myosin phosphatase and thus continued myosin light chain activity, during uterine contractility. The aim of this study therefore, was to investigate the effect of obesity on the expression of various components of the RhoA/ROCK pathway in human myometrium at term pregnancy.

**Methods:**

Protein was isolated from myometrial biopsies obtained at elective caesarean section, at term pregnancy from obese women and from those with a normal body mass index. Western blotting was performed using specific primary antibodies to RhoA/ Rho kinase associated proteins.

**Results:**

The protein expression of p160 ROCK-1 was significantly decreased (*P* < 0.001) in the myometrium from women in the obese cohort (*n* = 22) at term pregnancy, compared to women of those of normal body mass index (*n* = 15). No alteration in expression of the other proteins investigated was noted.

**Conclusions:**

The significant decrease in p160 ROCK-1 protein expression observed in the myometrium of obese women at late gestation may contribute to an inhibitory effect on contractility at labour, due to its contribution to calcium sensitisation and possibly other signalling pathways. These findings are relevant to the concept of compromised myometrial function in obese parturients.

## Background

Obesity is a big health problem in society, and is an increasing one in obstetric practice [1]. A wide variety of perinatal complications are associated with maternal obesity including prolonged pregnancy, less likelihood of spontaneous onset of labour at term, slower cervical dilation, longer duration of labour and an increased risk of intrapartum caesarean delivery [2,3]. Increased maternal body mass index (BMI) has also been significantly associated with ineffective uterine contractility [4], and the reasons for this are complex, and have been linked to elevated cholesterol levels (6) and a reduction in intracellular free calcium [Ca^(2+)^] flux in previous reports [5]. These findings suggest a possible dysregulation in myometrial contractility pathways occurs in association with maternal obesity, but there are minimal data to clearly support this hypothesis or explain the potential mechanisms. Adipocytokines have been postulated as having a metabolic role which exerts an inhibitory effect on myometrium in obese parturients. Our group has highlighted that leptin along with other adipocytokines, apelin and ghrelin inhibit uterine contractility *in vitro*[1,6-8]. However the potential signalling pathways that are involved are unknown.

Uterine smooth muscle contraction is primarily regulated by an increase in intracellular free calcium ([Ca^2+^]_i_), which leads to the activation of calmodulin-dependent myosin light chain kinase (MYLK). MYLK phosphorylates the regulatory myosin light chain (MLC), enhancing actin-myosin ATPase activity to cause contraction [9]. Dephosphorylation of the phosphorylated MLC by myosin phosphatase (MYPT) results in relaxation [10]. Meanwhile, MYPT is inhibited by phosphorylation by the protein kinase, Rho (Ras homolog gene family, member) -associated, coiled-coil containing protein kinase 1 (ROCK-1) at positions Thr696 or Thr853, either simultaneously or independently [11,12]. MYLK is activated by Ca^2+^-calmodulin whereas MYPT is inhibited by phosphorylation by Ca^2+^-independent mechanisms, leading to generation of increased MLC phosphorylation and force for a given intracellular Ca^2+^ concentration, a phenomenon known as ‘calcium-sensitisation’ [13]. Human pregnant myometrium expresses Rho regulatory proteins [14], including ROCK-1 and 2 [15,16]. There seems to be no significant change in the expression of ROCK-1 or 2 with pregnancy or labour [15-17]. However, in leptin-receptor deficient mice there is an altered contribution of RhoA/ROCK-1 signalling in the contractile activity of myometrium, suggesting leptin suppression of myometrial ROCK-1 and −2 expression and function, resulting in an inability to generate tonic contractions needed for delivery [18]. We therefore hypothesised that the RhoA/ROCK pathway may be linked to abnormal myometrial contractility in obese pregnant women. The aim of this study was to determine the effect of obesity in pregnancy on the expression of Rho and ROCK associated proteins in myometrium at term gestation in obese pregnant women, and in a control group of women with normal BMI.

## Methods

### Tissue samples

Biopsies were obtained at elective caesarean section in the third trimester of pregnancy in the Department of Obstetrics and Gynaecology, University College Hospital, Galway, Ireland. Ethical committee approval for tissue collection was obtained from the Research Ethics Committee at University College Hospital Galway and recruitment of patients was by informed written consent. The BMI calculation for women recruited was based upon weight and height measurements obtained at the first antenatal visit. Normal BMI was classified as 18.5 to 24.9 kg/m^2^ and the obese category BMI was classified as 30 kg/m^2^ or greater. The biopsy specimens were excised from the upper portion of the lower segment of the uterus. Myometrial samples were carefully dissected to minimise decidual inclusion. Immediately upon collection, the tissue was snap-frozen in liquid nitrogen in the operating theatre and stored at −80°C.

### Study patients

Biopsies were obtained at elective caesarean section (*n* = 37). The reasons for elective caesarean section in the normal BMI cohort (*n* = 15) included as follows: 1 previous caesarean section (*n* = 4), 2 previous caesarean sections (*n* = 5), breech presentation (*n* = 3), Intrauterine growth restriction (IUGR) (*n* = 1), polyhydraminos (*n* = 1) and previous endometrial ablation (*n* = 1). The reasons for elective caesarean section in the obese category (*n* = 22) were as follows: 1 previous caesarean section (*n* = 8), 2 previous caesarean sections (*n* = 3), 3 previous caesarean sections (*n* = 1), breech presentation (*n* = 5), previous vaginal surgery (*n* = 1), congenital hearing impairment (*n* = 1), foetal macrosomia (*n* = 1), previous myomectomy (*n* = 1) and previous third degree perineal tear (*n* = 1) The patient demographic data for both sets of samples is presented in Tables 1 and 2.

**Table 1:** **Patient Demographics of women with a BMI of 18.5 - 24.9 kg/m**^**2 **^**(*n *= 15)**

	**Age (yrs)**	**Gestation (wks)**	**Baby wt. (kg)**	**BMI value**
*Mean +/− SEM*	36.5 +/− 1.15	38.5 +/− 0.2	3.3 +/− 0.1	24.0 +/− 0.3
*Range*	29 - 45	37 - 41	2.6 – 4.2	21.7- 24.9

**Table 2:** **Patient Demographics of women with a BMI of ≥ 30 kg/m**^**2 **^**(*n *= 22)**

	**Age (yrs)**	**Gestation (wks)**	**Baby wt. (kg)**	**BMI value**
*Mean* +/− *SEM*	32.4 +/− 1.1*	39.0 +/− 0.1	3.6 +/− 0.1*	33.8 +/− 0.9***
*Range*	19 - 39	35 - 41	2.6 - 4.7	30.3 – 49.6

*** indicates a significance value of *P* < 0.001, * indicates a significance value of *P* < 0.05, compared to the normal BMI group.

### Protein isolation

Human myometrial tissue was disrupted in liquid nitrogen using a pestle and mortar prior to homogenisation in ice cold lysis buffer [19] containing 10 mM HEPES pH 7.5, 10 mM MgCl2, 5 mM KCL, 0.1% Triton X-100, 0.1 mM EDTA pH 8 (Sigma Aldrich, Dublin, Ireland) and a complete mini-protease inhibitor tablet (Hoffmann-La Roche Ltd., Basel, Switzerland) [20]. Cellular debris was removed by centrifugation at 5,000 × RPM, at 4°C for 10 minutes. 5 X Laemmli buffer containing 50 mM DTT was added to the supernatant, samples were used or stored at −80°C.

### Protein gel electrophoresis

The protein preparation was resolved by electrophoresis on Mini-PROTEAN TGX precast 10% or 12% (Biorad, Hercules, CA, USA) SDS (w/v) polyacrylamide gel electrophoresis (SDS-PAGE) gels at 30–50 Volts (V) at room temperature for 1–2 hours (BioRad, USA) in a buffer containing 25 mol l^-3^ Tris base, pH 8.3, 19.2 mol l^-2^ glycine, and 0.1% (w/v) SDS. The separated proteins were transferred to Immobilon-P transfer membranes (Millipore, Billerica, MA, USA) at a constant voltage of 25 V at 4°C overnight in transfer buffer containing 25 mol l^-3^ Tris base, 192 mol l^-3^ glycine, and 20% methanol (v/v), high-performance liquid chromatography (HPLC) grade (Sigma-Aldrich, Dublin, Ireland). To ensure transfer and equal loading of proteins, blots were stained with Ponceau S solution (Sigma-Aldrich, Ireland) for 5 minutes, followed by washing with de-ionised water. Membranes were blocked upon incubation for 1 hour at room temperature with phosphate-buffered saline (PBS; 1 mol l^-2^ phosphate buffer, 2.7 mol l^-3^ potassium chloride, and 1.37 mol l^-1^ sodium chloride, pH 7.4) containing 0.05% Tween 20 (v/v) (Sigma-Aldrich, Ireland) and 5% low-fat milk powder (w/v) (Carnation Milk, Nestlé S.A., Vevey, Switzerland) to block non-specific binding.

### Western blot analysis

Blots were incubated overnight in 1 X PBS containing 0.05% Tween 20 and 5% milk with the primary antibody at 4°C. The following primary antibodies (1/50 - 1/500 dilution) were utilised: anti-human ROCK-1 mouse polyclonal primary antibody (G-6 sc17794), ROCK-2 rabbit polyclonal G6 (sc5561), RhoA mouse monoclonal (26C4 sc418), caveolin-1 rabbit polyclonal (N-20 sc894), Rho8 (Rnd3) mouse monoclonal (clone 4 sc53874), moesin goat polyclonal (C-15 sc6410), pmoesin Thr558 goat polyclonal (sc12895), OTR rabbit polyclonal (H-60 sc33209), RhoGDI rabbit polyclonal (A20 sc360), MYPT 1 rabbit polyclonal (H-130 sc25718), MYL-9 (MLC) (D-15 sc-34487), ppMLC 18/19 goat polyclonal (sc12896 Santa Cruz Biotechnology Inc., Rockford, IL, USA), pMYPT 853 rabbit (Upstate, Millipore, Billerica, MA, USA), pMYPT Thr696 rabbit polyclonal (Upstate Millipore, USA ABS454), MLC mouse monoclonal (ab97981 Abcam plc, Milton Road, Cambridge, UK), RhoE (Rnd3) mouse monoclonal 3664 (Cell Signaling Technology, Danvers, MA, USA), caspase 3 monoclonal rabbit (Cell Signaling, USA 8G10 9665), cleaved caspase 3 rabbit monoclonal (Cell Signaling, USA Asp175 5A1E), calponin mouse monoclonal (DakoCytomation Ltd, UK M3556), pMAPK mouse monoclonal antibody to ERK1 and 2 pT185 and Y187 (Abcam, UK 50011) or 1/10,000 dilution of β-actin (ACTB) mouse monoclonal AC-15 (Sigma-Aldrich, Ireland) antibodies. Blots were washed and incubated in either 1/1,000 dilution of donkey anti-goat (Pierce Technology, Hallandale, FL., USA), 1/1000 goat anti-rabbit (Pierce Technology, USA), or 1/5000 dilution anti-mouse (Millipore, USA) horseradish peroxidase-conjugated secondary antibodies, the bound secondary antibody was detected as previously described. The membranes were then scanned with the fluorescence imager (FluorchemTM 8900, Alpha Innotech Corporation, San Leandro, California, USA) and AlphaEaseFC software was used to detect the signal, the image was processed and protein expression levels were determined by densitometric analysis compared to corresponding levels of the housekeeping protein, β-Actin [8].

### Statistical analysis

Statistical 2-tailed unpaired student t-tests were performed on the means of each group in order to assess the differences between groups, and graphs were constructed using GraphPad Prism version 4 (GraphPad Software Inc., USA). The mean, significance (*P*) values, standard error of the mean (SEM) and range values are presented in the text. *P* values < 0.05 were considered to be statistically significant.

## Results

### Patient demographics

The demographic details of all the patients in this study (*n* = 37) are contained in Tables 1 and 2. We statistically analysed the p160 ROCK-1 expression results in relation to maternal age alone; the mean ± SEM for women of less than 35 years (*n* = 14) was 0.71 ± 0.05, the mean for women aged 35 years and over (*n* = 23) was 0.89 ± 0.07 (*P* = 0.08). Parity for those in the normal BMI group was 0: 6 women, 1: 8 women and ≥2: 1 woman and for those in obese group, 0: 13 women, 1: 8 women and ≥2: 1 woman.

### Western blots

**Figure 1:**
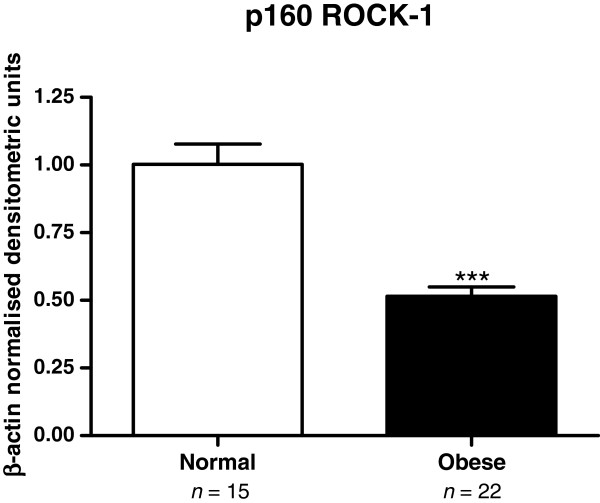
**A graph of the densitometric analysis of p160 ROCK-1 protein expression in human myometrium in women at term pregnancy, with a normal BMI (*n *= 15), compared to those in the obese cohort (*n *= 22).** The graph represents β-Actin normalised densitometric units of p160 ROCK-1 protein plotted against BMI values ± SEM (indicated with error bars). *** indicates a significance value of *P* < 0.001.

The expression of p160 ROCK-1 protein was significantly reduced in the myometrium from women in the obese category (*n* = 22) compared to women from the normal BMI group (*n* = 15) (Figure 1). The densitometric mean ± SEM of the normal BMI group was 1.003 ± 0.0075 (range 0.72 - 1.66) compared to 0.4912 ± 0.033 for the obese group (range 0.19 - 0.73), *P* < 0.001.

**Figure 2:**
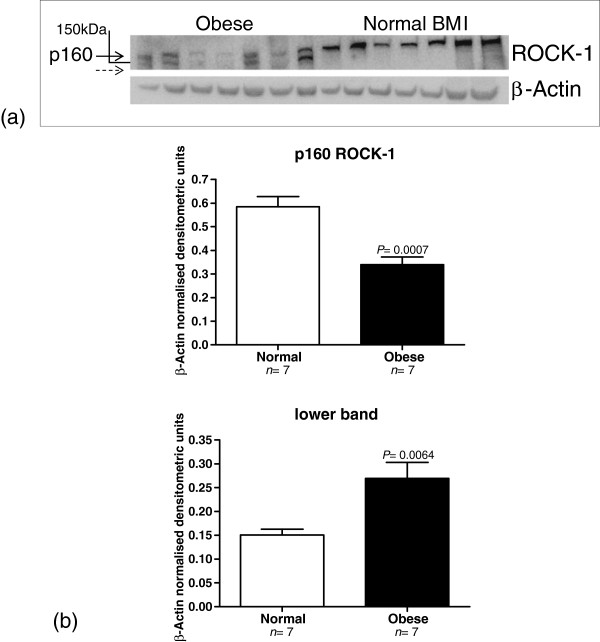
**A representative western blot of p160 ROCK-1 protein expression in myometrium from women with normal BMI (*n *= 7) compared to those in the obese category (*n *= 7).** The top band in the first blot indicated with the arrow is p160 ROCK-1 while the lower unidentified band, indicated with the broken arrow, was also quantified. The corresponding β-Actin western blot is also presented. The molecular weights (kDa) are represented by a line **(a)**. Quantitative densitometric analyses of the expression of the p160 ROCK-1 and the lower band are presented below the western blots, with β-Actin-normalised densitometric units plotted against BMI values, at term pregnancy ± SEM (indicated with error bars). The significance values (*P*) are indicated **(b)**.

In a representative Western blot, the expression of p160 ROCK-1 was demonstrated to be significantly decreased in the myometrium from women in the obese category (*n* = 7), compared to women from the normal BMI cohort (*n* = 7) (*P* = 0.0007) (Figure 2). The expression of a lower band in the ROCK-1 western blots was also observed to be increased in the samples that demonstrated a significant reduction in p160 ROCK-1 protein expression. In this experiment the expression of the lower band was observed to significantly increase in the same obese samples as those above (*P* = 0.0064) (Figure 2).

The mean densitometric units for the expression of p160 ROCK-1 in the normal BMI group was 0.584 ± 0.044, the range was 0.44 to 0.76. The mean densitometric value for the p160 ROCK-1 protein in the high BMI group was 0.34 ± 0.032 (range 0.22 - 0.49).

The mean value for the lower band in the p160 ROCK-1 blot for the normal BMI group was 0.151 ± 0.012 (range 0.11 to 0.185). The mean expression value of the lower band in the high BMI group was 0.269 ± 0.034 (range 0.16 - 0.39).

**Figure 3:**
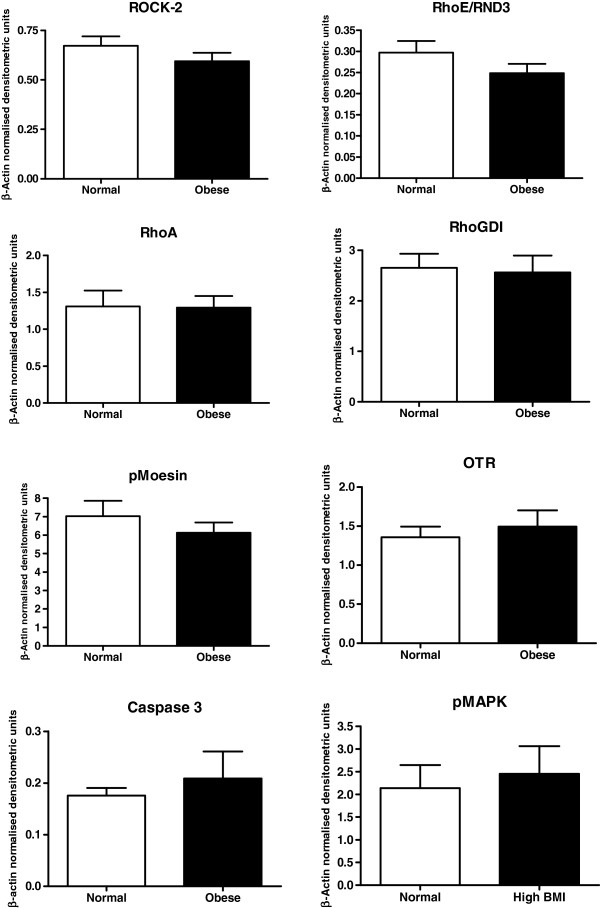
**Graphs of the densitometric analyses of the expression of RhoA/ROCK associated proteins, ROCK-2, RhoE, RhoA, RhoGDI, OTR in human myometrium from women with normal BMI (*n *= 15), compared to those in the obese category (*n *= 22), and caspase 3, pmoesin and pMAPK (*n *= 7 for both groups).**

**Figure 4:**
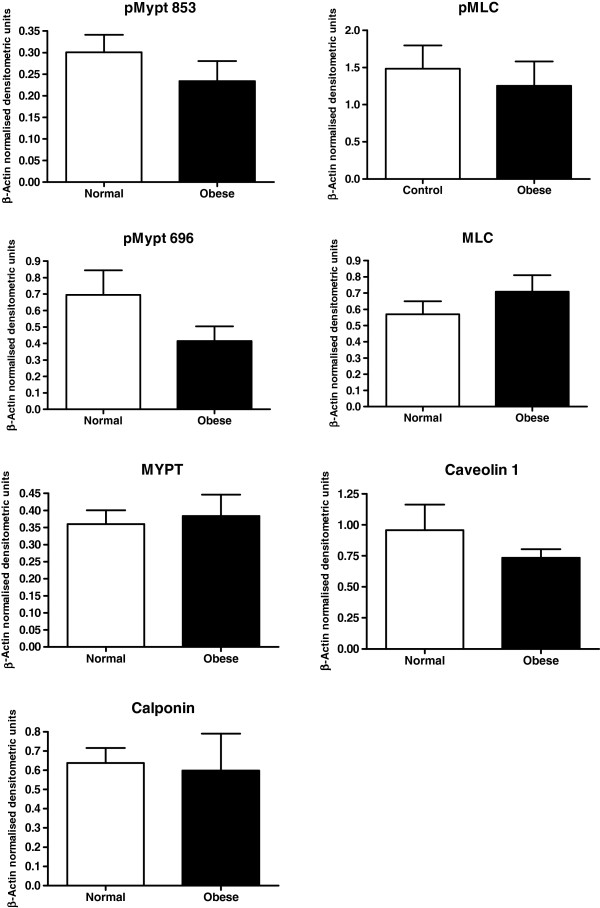
**Graphs of the densitometric analyses of the expression of RhoA/ROCK associated proteins, (p)(p)MYPT and (pp)MLC in human myometrium from women with normal BMI (*n *= 15), compared to those in the obese category (*n *= 22), and calponin and caveolin-1 (*n *= 7 for both groups).**

There were a series of proteins for which expression was detected in samples but there was no alteration thereof noted between the myometrium from the women of normal BMI, and that obtained from women in the obese category BMI. A list of these proteins is as follows: RhoA, Rho kinase 2 (ROCK-2), Rho guanine nucleotide dissociation inhibitor (RhoGDI), both phosphorylated and unphosphorylated myosin light chain (MLC and ppMLC 18/19), phosphorylated and unphosphorylated myosin phosphatase (MYPT and pMYPT 696 and pMYPT 853), Rho family protein Rnd3, oxytocin receptor (OTR), phosphorylated mitogen-activated protein kinase (pMAPK), caspase 3, phosphorylated moesin (pMoesin), caveolin-1 and calponin. This is demonstrated in Figures 3 and 4.

## Discussion

The findings from this study demonstrate a decrease in p160 ROCK-1 protein expression in the myometrium of obese pregnant women at term. This is an interesting finding considering the putative dysfunctional uterine activity that is believed to occur in obese pregnant women undergoing labour. The reasons for, or significance of, this finding are unclear but it is tempting to speculate that such reduced expression might exert a negative effect on uterine contractility in obese women, given the role of p160 ROCK-1 in calcium sensitisation, and also the roles it plays in other signalling pathways linked to contractility [10,12]. The strengths of this study are that the experiments were carried out in a relatively large number of samples, the clinical and BMI data are all reliable, the samples were collected and frozen freshly, there was no apparent difference in relation to maternal age, and there was a large difference in the expression levels observed. The limitations of this study include the fact that protein expression was investigated, but not protein activity, and that all samples were obtained prior to the onset of labour. In addition we have not related the findings observed in a functional model. The potential signalling pathways that may mediate the effects of such reduced expression are many, and this would require an extensive series of pharmacological experiments, which is a topic for further study.

As a consequence of the finding of reduced expression of p160 ROCK-1 in myometrium from pregnant women at term, and the suggestion that this may compromise the capacity of the myometrium to contract adequately, we subsequently examined the expression of other members of the RhoA/ROCK pathway. There was no change in RhoA, ROCK-2, Rnd3, calponin, OTR or caveolin-1 protein expression in association with increased maternal BMI. Similarly no change was observed in myometrial MYPT and MLC protein expression and phosphorylation, or the phosphorylation of the ROCK effector proteins moesin and MAPK. It is difficult to propose an overall mechanism of effect linked to the reduced p160 ROCK-1 expression observed, but the concept remains that it may be clearly linked to reduced myometrial contractility.

What could be responsible for the decrease in ROCK-1 expression? One possibility is that secretory factors produced by adipose tissue of overweight and obese women may alter the myometrial physiology. More than 100 substances including adipocytokines are secreted from the adipose tissue [21]. Interestingly, there is an altered contribution of RhoA/Rho kinase signalling in contractile activity of myometrium in leptin receptor-deficient mice. The authors of this latter study demonstrated that leptin suppressed myometrial ROCK-1 and 2 expression and function, resulting in an inability to generate tonic contractions needed for delivery [18]. Thus, it is possible that increased leptin levels in obese pregnant women might be in part responsible for the reduction in ROCK-1 protein expression, and may result in reduced myometrial contractility.

Another possibility in consideration of the results of this study is that p160 ROCK-1 protein reduction occurs, secondary to protein cleavage. The appearance of the lower band in the ROCK-1 western blots prompted us to investigate protease involvement in the decline of the p160 ROCK-1 protein. Elevated caspase 3 levels have been noted in pregnant mouse, rat and human uterus where it is thought to be involved in the regulation of uterine quiescence [22-24]. We did detect Caspase 3 protein in the myometrial biopsies however there was no significant change in expression and no observable cleaved caspase-3 protein. Indeed, phosphorylation of p160 ROCK-1 or other biochemical mechanisms may be responsible for the additional band. However, further investigation is necessary on this matter.

## Conclusions

ROCK-1 plays a central role in myometrial contractility and is closely linked to other signalling molecules in the cell. The reduction in its expression demonstrated in this study in the myometrium of obese pregnant women at term may play a role in inhibiting contractility in these women.

## Competing interests

The authors’ declare that they have no competing interests.

## Authors’ contributions

MOB conceived the idea and design of the project, collected myometrial biopsies, isolated protein, performed the western blots, did the statistical analysis and drafted the manuscript. SC collected myometrial biopsies and prepared tissue protein. JJM provided access to the myometrial tissue samples and patient data, and edited the final document. TJS acquired funding. All authors read and approved the final manuscript.
